# Significant association of elevated serum galectin-9 levels with the development of non-alcoholic fatty liver disease in patients with rheumatoid arthritis

**DOI:** 10.3389/fmed.2024.1347268

**Published:** 2024-02-02

**Authors:** Po-Ku Chen, Wei-Fan Hsu, Cheng-Yuan Peng, Tsai-Ling Liao, Shih-Hsin Chang, Hsin-Hua Chen, Chu-Huang Chen, Der-Yuan Chen

**Affiliations:** ^1^Rheumatology and Immunology Center, China Medical University Hospital, Taichung, Taiwan; ^2^Translational Medicine Laboratory, China Medical University Hospital, Taichung, Taiwan; ^3^College of Medicine, China Medical University, Taichung, Taiwan; ^4^Center for Digestive Medicine, Department of Internal Medicine, China Medical University Hospital, Taichung, Taiwan; ^5^Ph.D. Program in Translational Medicine and Rong Hsing Research Center for Translational Medicine, National Chung Hsing University, Taichung, Taiwan; ^6^Department of Medical Research, Taichung Veterans General Hospital, Taichung, Taiwan; ^7^College of Medicine, National Chung Hsing University, Taichung, Taiwan; ^8^Division of General Medicine, Department of Medicine, Taichung Veterans General Hospital, Taichung, Taiwan; ^9^Vascular and Medicinal Research, Texas Heart Institute, Houston, TX, United States; ^10^Institute for Biomedical Sciences, Shinshu University, Nagano, Japan

**Keywords:** non-alcoholic fatty liver disease, galectin-9, soluble T cell immunoglobulin and mucin-containing-molecule-3 (sTIM-3), fatty acid-binding proteins, rheumatoid arthritis

## Abstract

**Background:**

Non-alcoholic fatty liver disease (NAFLD) is prevalent among rheumatoid arthritis (RA) patients, but its pathogenesis has rarely been explored. Galectin-9 (Gal-9) interacts with T cell immunoglobulin and mucin-containing-molecule-3 (TIM-3) expressed on hepatocytes and thus regulates T cell proliferation in a murine model of NAFLD. We aimed to examine the pathogenic role of the Gal-9/TIM-3 pathway in RA-NAFLD.

**Methods:**

Serum levels of Gal-9, soluble TIM-3 (sTIM-3), fatty acid-binding proteins (FABP)1, and FABP4 were determined by ELISA in forty-five RA patients and eleven healthy participants. Using Oil-red O staining and immunoblotting, we examined the effects of Gal-9 and free fatty acid (FFA) on lipid accumulation in human hepatocytes and FABP1 expression.

**Results:**

Serum Gal-9, sTIM-3 and FABP1 level were significantly higher in RA patients (median 5.02 ng/mL, 3.42 ng/mL, and 5.76 ng/mL, respectively) than in healthy participants (1.86 ng/mL, 0.99 ng/mL, and 0.129 ng/mL, all *p* < 0.001). They were also significantly higher in patients with moderate-to-severe NAFLD compared with none-to-mild NAFLD (*p* < 0.01; *p* < 0.05; and *p* < 0.01, respectively). Serum Gal-9 levels were positively correlated with sTIM-3, FABP1, FABP4 levels, and ultrasound-fatty liver score, respectively, in RA patients. Multivariate regression analysis revealed that Gal-9 (cut-off>3.30) was a significant predictor of NAFLD development, and Gal-9 and sTIM-3 were predictors of NAFLD severity (both *p* < 0.05). The cell-based assay showed that Gal-9 and FFA could upregulate FABP1 expression and enhance lipid droplet accumulation in hepatocytes.

**Conclusion:**

Elevated levels of Gal-9 and sTIM3 in RA patients with NAFLD and their positive correlation with NAFLD severity suggest the pathogenic role of Gal-9 signaling in RA-related NAFLD.

## Introduction

Non-alcoholic fatty liver disease (NAFLD), the most common cause of chronic non-viral liver disease in Western countries (1), is characterized by the presence of steatosis in more than 5% of hepatocytes occurring in individuals with minimal or no alcohol consumption (1). NAFLD represents histological changes ranging from indolent and simple steatosis to nonalcoholic steatohepatitis (NASH) (1) and may progress to cirrhosis, liver failure, and hepatocellular carcinoma (2, 3). Rheumatoid arthritis (RA), an inflammatory autoimmune disease characterized by chronic synovitis and bone destruction, is often associated with poor life quality (4). Several epidemiology studies observed a high prevalence of NAFLD-related risk factors (e.g., metabolic syndrome (MetS), dyslipidemia, and type II diabetes mellitus (DM)) in RA patients (5–8). The use of conventional synthetic disease-modifying anti-rheumatic drugs (csDMARDs) may also add to more NAFLD risk in RA patients (9, 10). Accordingly, an estimated 20.3–30.0% of RA patients would be affected by NAFLD (11, 12).

Although liver biopsy is considered the gold standard for staging NAFLD (13), it is not always clinically applicable due to its invasiveness and risk of complications (14). Hence, a non-invasive assessment using markers for NAFLD is worth investigating. Galectins are a group of mammalian lectins that have a high affinity for β-linked galactose residues and share a highly conserved carbohydrate recognition domain (CRD) (15). Galectin-9 (Gal-9), a ligand of T cell immunoglobulin and mucin-containing-molecule-3 (TIM-3) is expressed on type 1 helper T (Th1) and Th17 cells and provides inhibitory signals (16). Gal-9 regulates pro-inflammatory T-cell responses through the Gal-9/TIM-3 pathway and induces apoptosis of Th1 or Th17 cells (17). Increasing evidence indicates that Gal-9 is highly expressed in RA-derived synovial tissues and peripheral blood T cells (18, 19) and may promote angiogenesis and joint inflammation (20). Compared to healthy participants, RA patients had lower frequencies of CD56^dim^ natural killer (NK) cells (21), which were also observed in NAFLD patients (22). Besides, elevated Tim-3 expression in various T cell subsets and monocytes correlates with the disease activity of RA patients (23). Moreover, Tang et al. demonstrated that Gal-9 could interact with TIM-3 expressed on hepatocytes and thus regulate CD4^+^NKT cells proliferation in the murine model of NAFLD (24). These findings suggest that Gal-9 and TIM-3 may partake in the pathogenesis of RA-related NAFLD, which has yet to be explored.

Fatty acid-binding proteins (FABPs) are cytosolic proteins essential for the binding of hydrophobic ligands such as fatty acids (25, 26). Liver FABP, also known as FABP1, is specifically and highly expressed in hepatocytes and accounts for approximately 5% of cytosolic proteins (27). It contributes to regulating lipid metabolism, fatty acid oxidation, lipotoxicity, and oxidative stress (28, 29). In the liver of mice with FABP1 silencing (30) and FABP1-deficient mice with NAFLD (31), deficient FABP1 expression not only led to weight decline and decreased triglyceride content in the liver but also suppressed the expression of hepatic inflammatory cytokines. FABP4, or adipocyte FABP, is highly expressed in adipocytes and may contribute to insulin resistance and atherosclerosis by functioning along both metabolic and inflammatory pathways (32). Thus, serum FABP1 and FABP4 have been used as diagnostic markers for NAFLD (33, 34).

In this pilot study, we compared the differences in serum levels of Gal-9, sTIM-3, FABP1, and FABP4 between RA patients and healthy participants, and between RA patients with none-to-mild and moderate-to-severe NAFLD. We also examined the associations between serum Gal-9 and each of the following: sTIM-3, FABP1, FABP4, and the NAFLD severity estimated by abdominal sonography in RA patients. The change in serum Gal-9 and FABP1 levels was also evaluated in RA patients after 12 months of therapy.

## Methods

### Patients

In this single-center and prospective study, we consecutively recruited 45 patients who met the 2010 revised criteria of the American College of Rheumatology (ACR)/European League Against Rheumatism (EULAR) collaborative initiative for RA (35). All patients had received csDMARDs, including methotrexate (MTX), before the use of biologic/targeted synthetic DMARDs (b/tsDMARDs). The exclusion criteria were mainly current infection, malignancy, or the detectable viral loads for hepatitis B or hepatitis C. Disease activity was assessed using the 28-joint disease activity score-erythrocyte sedimentation rate (DAS28-ESR) (36). According to the guidelines of the British Society for Rheumatology (37), these patients received b/tsDMARDs, including TNF-α inhibitors, non-TNF-α inhibitors, or Janus kinase inhibitors (JAKi). Eleven healthy volunteers who had no rheumatic disease were enrolled as control subjects. The Institutional Review Board of our hospital approved this study (CMUH108-REC3-113, CMUH110-REC3-187), and each participant’s written consent was obtained according to the Declaration of Helsinki.

### Determination of serum levels of Gal-9, soluble TIM3, FABP1 and FABP4

Ten milliliters of whole blood were collected in a BD Vacutainer™ (BD Biosciences, San Jose, CA, United States) and centrifuged at 2,000 rpm for 10 min. Serum samples were stored at-80°C until use. Gal-9 (Cat#DY2054), sTIM-3 (Cat#DY2365), FABP1 (Cat#DY9465), and FABP4 (Cat#DY3105) were analyzed according to the manufacturer’s instructions using the Duoset-ELISA kit (R&D Systems. Minneapolis, MN, United States) for the assays. Briefly, 96-well microplates are coated with 100 μL of the diluted capture antibody per well overnight at room temperature (RT) and then incubated with 1% BSA in PBS (reagent dilution, 200 μL) for 1 h at RT. Added 100 μL of sample per well (3-fold dilution in reagent diluent) and incubated at RT for 2 h. Added 100 μL of the detection antibody (diluted 1,000X) to each well and incubated at RT for 2 h, and then 100 μL of Streptavidin-HRP was added to each well at RT with incubation of 20 min and avoided in direct light. Subsequently, each well was washed with PBS containing 0.1% Tween20 using a manifold dispenser, and then 100 μL of TMB (3, 3′, 5, 5′-tetramethylbenzidine). Substrate Solution was added to each well with an incubation time of 10 min at RT. Finally, 50 μL of Stop Solution (H_2_SO_4_) was added, and absorbance was measured at 450 nm or 540 nm by the BioTek Synergy HT plate reader (BioTek Instruments, Winooski, VT).

### Calculation of NAFLD or liver fibrosis markers

Aspartate aminotransferase (AST)-to-platelet ratio index (APRI) is calculated by: (AST in IU/L)/(AST upper limit of normal in IU/L)/(platelets in 10^9^/L), which can be used to estimate the risk of significant fibrosis. Fibrosis-4 (FIB-4) is calculated by: (age in years x AST in IU/L)/(platelet count in 10^9^/L x √ALT in IU/L).

### Abdominal ultrasonographic examination

Abdominal ultrasonography (US) scanning was performed after an 8 h overnight fast by a well-trained examiner with a 4.00 MHz transducer and a high-resolution B-mode scanner (Siemens Medical Solutions, Mountain View, CA). The US measurements were performed by an experienced hepatologist (W-FH) with a good professional background who performed >8,000 times liver US before the study and was blinded to participants’ data. The severity of NAFLD was calculated using the modified US-fatty liver indicator (FLI) score (38, 39), which ranges from 0 to 5. The US-FLI is composed of five indicators: the absence (score 0) or presence (score 1) of liver-kidney contrast, posterior attenuation of the ultrasound beam, vessel blurring, difficult visualization of the gallbladder wall, and difficult visualization of the diaphragm. Because the liver-kidney contrast grade was subjective (mild, moderate, and severe contrast were graded as 1, 2, and 3 in the original score, respectively), we modified the liver-kidney contrast grade to the absence or presence of liver-kidney contrast. The subjects were then divided into two groups based on NAFLD severity according to the US-FLI score: none-to-mild NAFLD (score 0–2) and moderate-to-severe NAFLD (score 3–5).

### Cell culture and treatment

Samples of human hepatocytes (Cat#5200, ScienCell, Carlsbad, CA, United States) were purchased by Sciencell™ company. The cells were cultured in hepatocyte basal medium supplemented with 2% FBS (Cat#0025, ScienCell), 5 mL of hepatocyte growth supplement (HGS, Cat. No. 5252, ScienCell), and 5 mL antibiotic solution (penicillin/streptomycin, cat. 0503, ScienCell) and maintained at 37°C in a humidified atmosphere of 5% CO2 and 95% air. Human hepatocytes were seeded at 1 × 10^4^ cells/well in 24 well-culture plates. The medium was replaced with serum-free DMEM for 24 h. Human hepatic cells were treated with Gal-9 coincubation with/without OA (100 nM) or PA (200 nM) and OA/PA in DMEM with 2% serum for 24 h and then further were examined with Oil-red O staining and Western blotting.

### Western blotting for FABP1 expression in human hepatocytes

Total proteins were extracted from human hepatocytes lysates. The samples were separated by 12.5% SDS-PAGE and then transferred to PVDF membranes (Millipore, Billerica, MA, United States). The membrane was blocked with 5% milk in PBS with 0.1% Tween-20 (PBST) (Bionovas, Inc., Washington, DC, United States) for 30 min at RT and subsequently incubated with specific anti-human FABP1 antibody (Cat# GTX53712, GeneTex Inc. Irvine, CA, United States) and anti-GAPDH antibody (Cat# GTX100118, GeneTex Inc. Irvine, CA, United States) at 4°Covernight. After washing with PBST three times, the membranes were incubated with horseradish peroxidase-conjugated secondary antibody (GeneTex Inc. Irvine, CA, United States). Immunoreactive bands were visualized with a chemiluminescence detection system ECL (Millipore, Billerica, MA, United States). Band intensity was determined by Image J software. The protein levels of FABP1 were normalized to GAPDH.

### Oil red O staining

Human hepatocytes were washed with PBS twice and fixed in 4% paraformaldehyde for 15 min and incubated with Oil Red O working solution for another 30 min at room temperature, followed by decolorization with 60% isopropanol. After washing three times with ddH2O, the cells were counterstained with hematoxylin for 1 min. The cells were photographed under a light microplate reader (Bio-Tek, United States). By examining the digitized image of lipid droplets with a gray-level histogram, a threshold value was settled to differentiate lipid droplets from other components within the image. Subsequently, the image was converted into a binarization using this selected threshold value. The area of lipid accumulation on human hepatocytes was then measured using the identified threshold value within 5 randomly selected microscopic fields.

### Statistical analysis

The results were presented as the mean ± standard deviation (SD) or median (interquartile range). The Mann–Whitney U and Kruskal-Wallis with post-hoc Dunn test were used for between-group comparison of the levels of Gal-9, sTIM3, FABP1, FABP4, and clinical parameters. The correlation coefficient was obtained through the nonparametric Spearman’s rank correlation test. Wilcoxon signed-rank test was employed to compare the levels of Gal-9 and FABP1 during follow-up in RA patients after 6 months of therapy. A two-sided *p*-value<0.05 was considered statistically significant.

## Results

### Clinical characteristics of RA patients

The baseline characteristics of the enrolled 45 patients are illustrated in Table 1. Based on NAFLD severity, 32 (71.1%) patients had none-to-mild NAFLD, and 13 (28.9%) had moderate-to-severe NAFLD. The mean age of RA patients with moderate-to-severe NAFLD and those with none-to-mild NAFLD were 60.9 and 58.0 years, respectively. As expected, significantly higher triglyceride levels were observed in patients with moderate-to-severe NAFLD than in those with none-to-mild NAFLD. Significantly higher disease activity markers, including serum levels of C-reactive protein (CRP) and DAS28-ESR scores, were also found in patients with moderate-to-severe NAFLD than in those with none-to-mild NAFLD. Otherwise, there were no significant differences in the proportion of females, body mass index, disease duration, the positivity for rheumatoid factor (RF) or anti-citrullinated peptide antibodies (ACPA), APRI, FIB-4, the prescribed medications, or comorbidities between both groups. RA patients were older than healthy control (HC) participants (mean 58.8 years versus 50.1 years, *p* < 0.05) (Supplementary Table S1). There was also no significant difference in the proportion of females or body mass index between RA patients and HC participants.

**Table 1:** Demographic data, clinical characteristics, laboratory findings, and the used medications in rheumatoid arthritis (RA) patients with different severity of non-alcoholic fatty liver disease (NAFLD).^a^

Characteristics	RA with moderate-severe NAFLD (*n* = 13)	RA with none-mild NAFLD (*n* = 32)	*p*-value
Age at study entry, years	60.9 ± 13.9	58.0 ± 11.7	0.453
Female, *n* (%)	11 (84.6%)	26 (81.3%)	0.789
Disease duration, years	9.54 ± 3.26	9.34 ± 2.30	0.572
Body mass index, kg/m^2^	26.0 ± 2.9	24.5 ± 4.3	0.078
Total cholesterol, mg/dL	199.1 ± 42.1	202.6 ± 34.3	0.559
Triglyceride, mg/dL	182.5 ± 104.5**	107.1 ± 42.9	0.006
LDL-C, mg/dL	114.9 ± 33.7	116.4 ± 27.8	0.679
HDL-C, mg/dL	58.6 ± 14.4	62.8 ± 16.2	0.551
Atherogenic index, (%)	3.49 ± 1.08	3.37 ± 0.81	0.973
RF-positivity, *n* (%)	9 (69.2%)	20 (62.5%)	0.669
ACPA-positivity, *n* (%)	9 (69.2%)	17 (53.1%)	0.521
C-reactive protein, mg/dL	1.49 ± 1.40***	0.45 ± 0.86	0.001
DAS28 score at baseline	4.51 ± 1.48***	3.14 ± 0.78	0.001
APRI	0.56 ± 0.70	0.32 ± 0.13	0.121
FIB-4	3.31 ± 6.46	1.35 ± 0.61	0.224
Concomitant corticosteroids, mg/day	0.56 ± 0.89	0.28 ± 0.61	0.282
Concomitant csDMARDs, *n* (%)			
Methotrexate	11 (84.6%)	26 (81.3%)	0.789
Hydroxychloroquine	7 (53.8%)	17 (53.1%)	0.964
Sulfasalazine	4 (30.8%)	12 (37.5%)	0.669
The use of bDMARDs, *n* (%)	7 (53.8%)	16 (50.0%)	0.662
The use of JAK inhibitors, *n* (%)	6 (46.2%)	13 (40.6%)	0.734
Comorbidities, *n* (%)			
Hypertension	4 (30.8%)	10 (31.3%)	0.974
Diabetes mellitus	2 (15.4%)	3 (9.4%)	0.561
Current smoker, *n* (%)	1 (7.7%)	3 (9.4%)	0.857

a Data were expressed as mean ± SD, number (%), ACPA, anti-citrullinated peptide antibodies; DAS28, the 28-joint disease activity score; RF, rheumatoid factor; LDL-C, low-density lipoprotein cholesterol; HDL-C, high-density lipoprotein cholesterol; APRI, aspartate aminotransferase (AST)-to-platelet ratio index; FIB-4, FIB-4; csDMARDs, conventional synthetic disease-modifying anti-rheumatic drugs; bDMARDs, biological DMARDs; JAK, Janus kinase. Atherogenic index is the ratio of total cholesterol/HDL-C. Chi-square test was used to compare binary variables.

**p < 0.01, ***p < 0.001, moderate–severe in RA patients vs. none-mild NAFLD in RA patients as determined by Mann–Whitney test.

### The differences in serum levels of Gal-9, sTIM-3, FAPB1, and FABP4 between RA patients and HC participants

**Figure 1:**
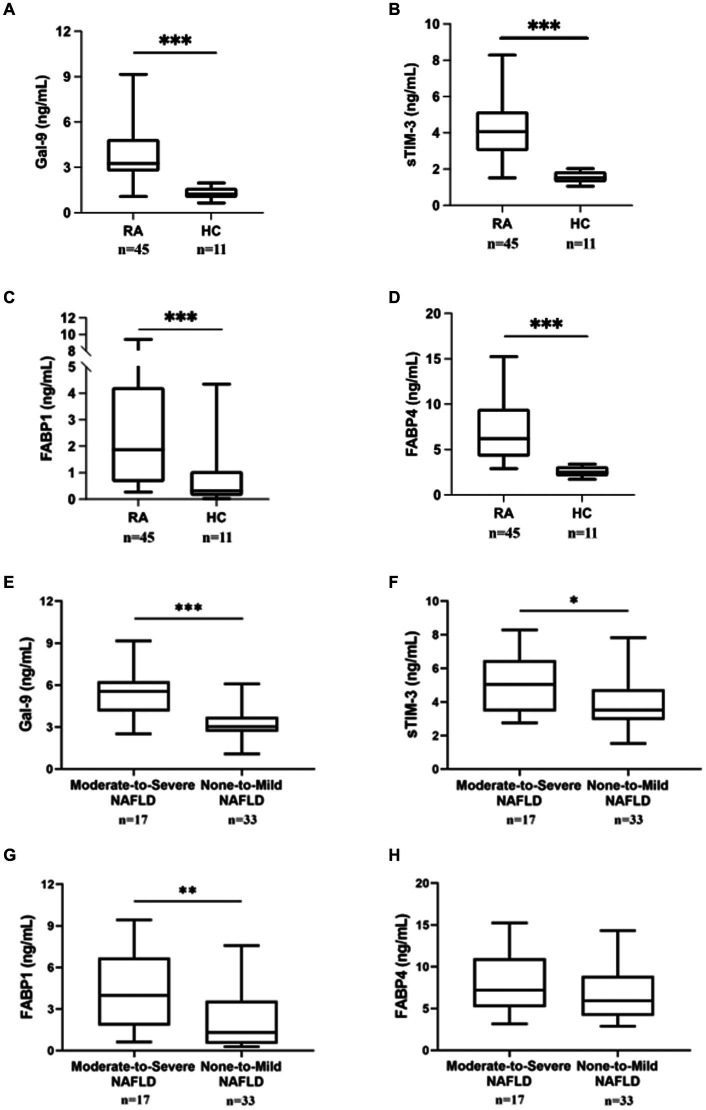
Comparing Gal-9, sTIM-3, FABP1, and FABP4 serum levels between RA patients with/without NAFLD and HC. Serum **(A)** Gal-9, **(B)** sTIM-3, **(C)** FABP1, and **(D)** FABP4 levels were expressed between RA patients and HC subjects. Serum **(E)** Gal-9, **(F)** sTIM-3, **(G)** FABP1, and **(H)** FABP4 levels expression in RA patients with severity NAFLD. RA subjects with fatty liver were diagnosed by ultrasound and then divided into two subgroups according to the severity of fatty liver: none-to-mild NAFLD (grades 0 to 2) and moderate-to-severe NAFLD (grades 3 to 5). Gal-9, galectin-9; sTIM-3, soluble a ligand of T cell immunoglobulin and mucin-containing-molecule-3; FABP, fatty acid binding protein. Data are presented as box-plot diagrams, with the box encompassing the 25th percentile (lower bar) to the 75th percentile (upper bar). The horizontal line within the box indicates median value, respectively, for each group. ^*^p < 0.05, ^**^p < 0.01, ^***^p < 0.001, determined by Mann–Whitney U test.

As shown in Figures 1A–D, serum levels of Gal-9, sTIM-3, FAPB1, and FABP*4* were significantly higher in RA patients (median 3.27 ng/mL, interquartile range [IQR] 2.72–4.87 ng/mL; 4.04 ng/mL, IQR 3.00–5.17; 1.87 ng/mL, IQR 0.66–4.23; 6.21 ng/mL, IQR 4.22–9.48, respectively) than in HC (1.24 ng/mL, IQR 1.00–1.29 ng/mL; 1.52 ng/mL, IQR 1.28–1.87 ng/mL; median 0.31 ng/mL, IQR 0.14–1.06; median 2.51 ng/mL, IQR 2.05–3.14, all *p* < 0.001, respectively).

### The differences in serum levels of Gal-9, sTIM-3, FAPB1, and FABP4 between RA patients with none-to-mild and moderate-to-severe NAFLD

Based on modified US-FLI (scores 0–5), RA patients were divided into two subgroups: 33 (66.0%) patients with normal liver function or mild NAFLD (scores 0–2) and 17 (34.0%) with moderate-to-severe NAFLD (scores 3–5). As shown in Figures 1E,F, RA patients with moderate-to-severe NAFLD had significantly higher serum levels of Gal-9 and sTIM-3 (5.55 ng/mL, IQR 4.14–6.27 ng/mL; 5.04 ng/mL, IQR 3.44–6.48 ng/mL, respectively) than those with none-to-mild NAFLD (3.04 ng/mL, IQR 2.68–3.73 ng/mL, *p* < 0.001; 3.51 ng/mL, IQR 2.94–4.75 ng/mL, *p* < 0.05, respectively). Serum FABP1 levels were also significantly higher in patients with moderate-to-severe NALFD (median 3.98 ng/mL, IQR 1.82–6.69 ng/mL) than in those with none-to-mild NAFLD (1.30 ng/mL, IQR 0.49–3.60 ng/mL, *p* < 0.01) (Figure 1G). In RA patients, there was no difference in FABP4 between moderate-to-severe NAFLD and none-to-mild NAFLD (Figure 1H).

### Correlation between Gal-9 levels and clinical laboratory data or between CRP and Gal-9 levels in RA patients with NAFLD

**Figure 2:**
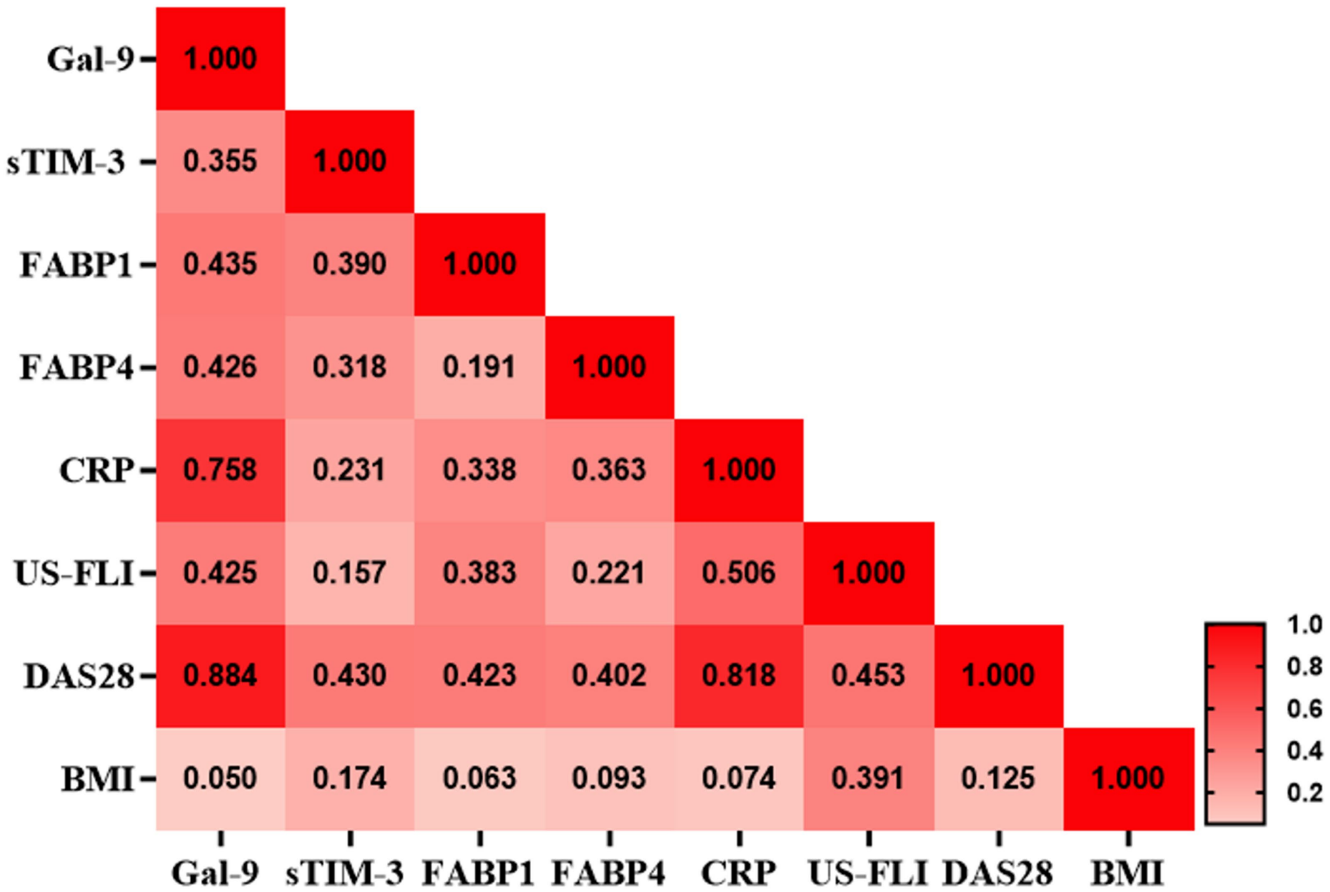
Correlation matrix between Gal-9 and inflammatory parameters in RA patients with NAFLD. Correlation matrix between Gal-9 and inflammatory parameters in RA patients with NAFLD. The correlation coefficient was obtained through the Spearman’s rank correlation test. CRP, C-reactive protein; Gal-9, galectin-9; FABP, fatty acid binding protein; FLI, US-fatty liver indicator; NAFLD, nonalcoholic fatty liver and RA, rheumatoid arthritis.

As shown in Figure 2, serum Gal-9 levels were positively correlated with sTIM-3 levels (*p* < 0.01), FABP1 levels (*p* < 0.01), FABP4 levels (*p* < 0.01), DAS28-ESR score (*p* < 0.001) and US-FLI score (*p* < 0.01), respectively, in RA patients. Besides, CRP levels were positively correlated with serum levels of Gal-9, FABP1, and FABP4 (Spearman r = 0.758, *p* < 0.001; r = 0.338, *p* < 0.05; r = 0.362, *p* < 0.05, respectively) in patients with RA.

### Gal-9 enhanced intracellular lipid accumulation on human hepatocytes

**Figure 3:**
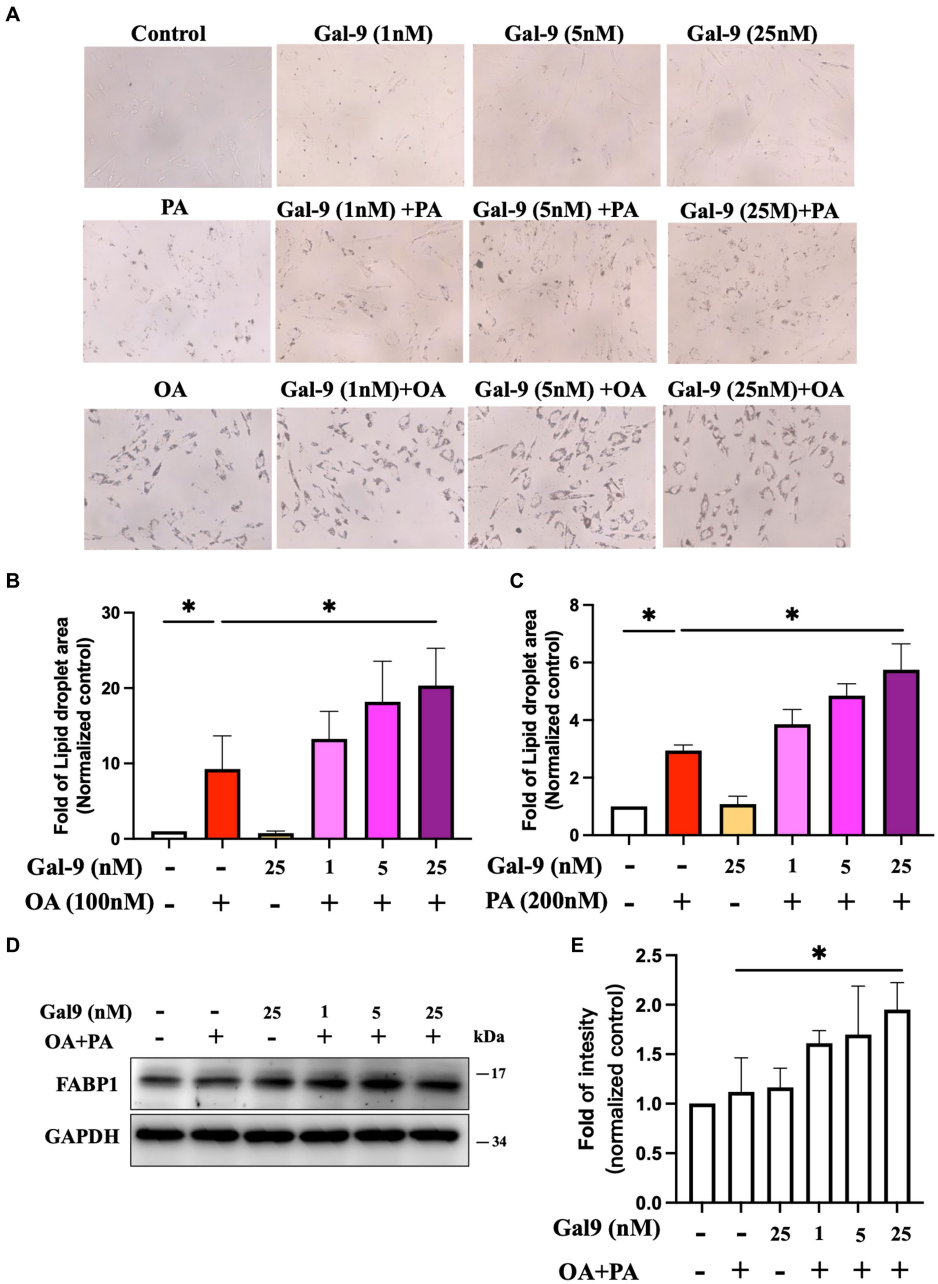
Effect of gal-9 on intracellular lipid accumulation in liver cells through inducing FABP1 expression. **(A)** Human hepatic cells were treated with Gal-9 coincubation with/without OA (100 nM) or PA (200 nM) for 24 h. The lipid droplet area of red oil O staining of human hepatocytes was measured using Image J software. The image was converted into a binarization using this selected threshold value. The area of lipid accumulation on human hepatocytes was then measured using the identified threshold value within 5 randomly selected microscopic fields. Fold change of normalized control was represented the three independent Red Oil O staining for **(B)** OA and **(C)** PA treatment. Fold change as mean ± SD. scale bar, 100 μm. These results were obtained in 3 independent experiments. **(D)** Gal-9 and OA/PA induced FABP1 expression in human hepatocytes. **(E)** Intensity of protein levels were quantified by Image J software for four independent western blotting. Data was represented as mean ± SD. Gal-9, galectin-9; FABP, fatty acid binding protein; OA, oleic acid; PA, palmitic acid. ^*^p < 0.05, determined by Mann–Whitney U test.

To investigate the influence of Gal-9 on free fatty acid (FFA)-induced steatosis, human hepatocytes were treated with FFA, including oleic acid (OA) or palmitic acid (PA), to induce steatosis, with or without preincubation with Gal-9 (40). Lipid droplets accumulated in hepatocytes were stained with oil-red O staining (Figure 3A). Gal-9 could significantly increase OA-and PA-induced lipid accumulation in hepatocytes compared to OA (Figure 3B) and PA (Figure 3C) treatment alone. FFA mixture (OA: PA = 2:1) can regulate FAPB1 expression that substantially traps lipids in the cells. We then examined the effect of Gal-9 on FABP1 expression and found that Gal-9 treatment could increase FAPB1 expression. Gal-9 and FFA mixture had dose-dependently synergistic effects on the upregulation of FABP1 expression (Figures 3D,E).

### Changes in serum levels of Gal-9 and FABP1 in RA patients after therapy

**Figure 4:**
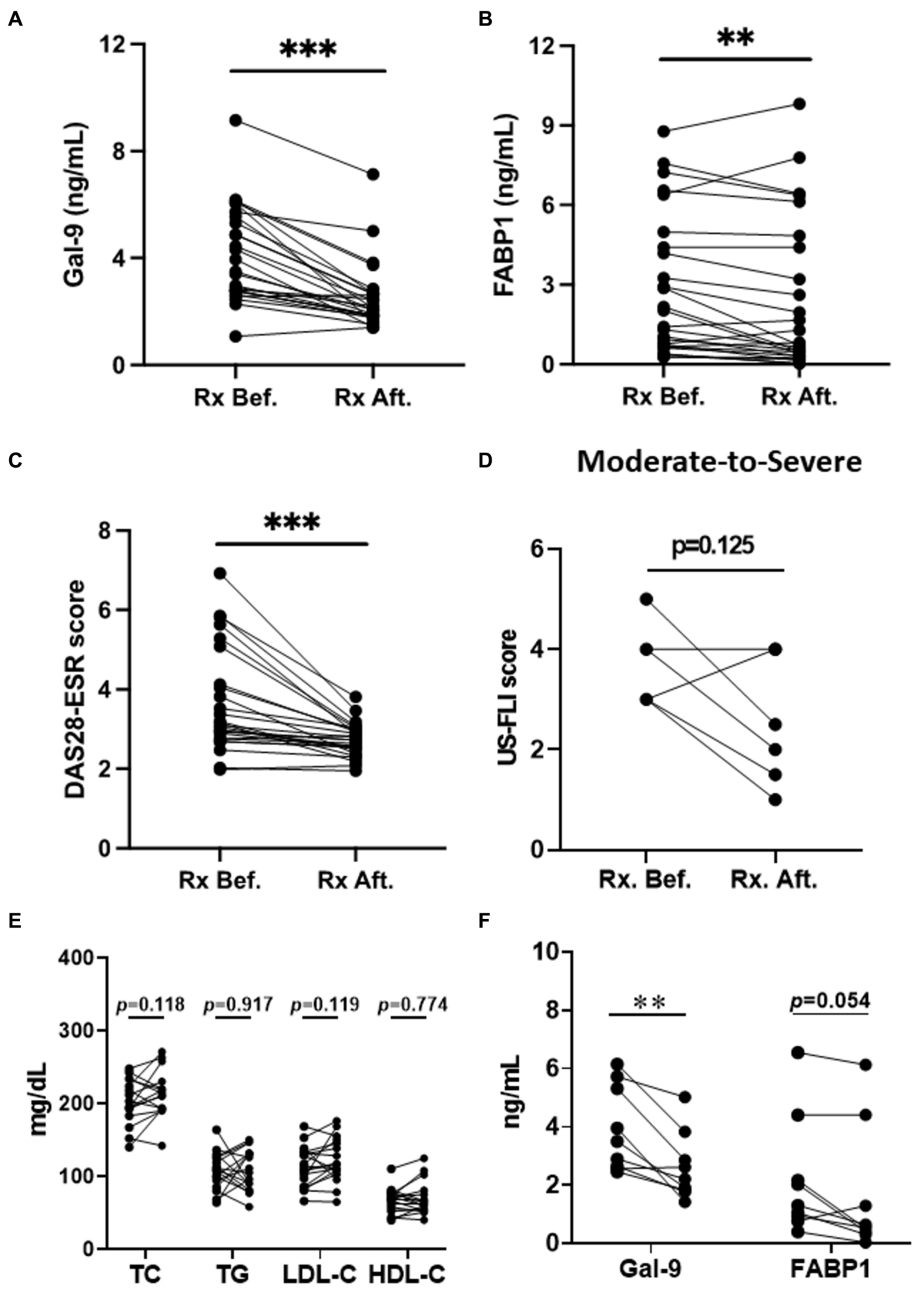
The change of Gal-9 levels, FABP1 levels, DAS28-ESR, US-FLI and Lipid profile in RA patents after therapy. **(A–C)** The change of Gal-9, FABP1 levels, and DAS28-ESR in RA patients with NAFLD after therapy. **(D)** The change of US-FLI in moderate-to-severe NAFLD in RA patients after therapy. **(E)** The change of Lipid profile in RA patients with JAKi treatment. **(F)** The change of Gal-9 and FABP1 in RA patients with JAKi treatment. Gal-9, galectin-9; FABP, fatty acid binding protein; NAFLD, nonalcoholic fatty liver; RA, rheumatoid arthritis. Disease activity was assessed using the 28-joint disease activity score-erythrocyte sedimentation rate (DAS28-ESR); US-fatty liver indicator (FLI). ***p < 0.001, determined by Wilcoxon signed rank test.

Twenty-six RA patients with NAFLD were available to examine their serum levels of Gal-9 and FABP1 both at baseline and after 12 months of treatment. As shown in Figure 4, serum Gal-9 (Figure 4A) and FABP1 (Figure 4B) levels significantly declined (3.72 ng/mL, IQR 2.78–5.59 ng/mL versus 2.10 ng/mL, IQR 1.84–2.67 ng/mL, *p* < 0.001; 1.72 ng/mL, IQR 0.66–4.55 ng/mL versus 0.77 ng/mL, IQR 0.31–4.51 ng/mL, *p* < 0.01, respectively), paralleling the reduction of RA disease activity, reflected by DAS28-ESR scores (Figure 4C) (mean ± standard deviation, 3.66 ± 1.31, 2.73 ± 0.42, *p* < 0.001) in patients receiving 12 months of b/tsDMARDs therapy. Although the severity of NAFLD reflected by the US-FLI score declined after b/tsDMARDs therapy in RA with moderate-to-severe NAFLD, it did not reach statistical significance (Figure 4D). A non-significant increase in TC, LDL-C, and HDL-C plasma levels in RA patients receiving 12 months JAKi treatment (Figure 4E). Gal-9 levels were significantly reduced (3.04 ng/mL, IQR 2.18–3.91 ng/mL versus 2.09 ng/mL, IQR 1.58–2.60 ng/mL, *p* < 0.001), and so were FABP1 levels (*p* = 0.054), after 12 months of JAKi therapy in RA patients (Figure 4F).

### Logistic regression analysis for predicting the emergence of NAFLD in RA patients

As illustrated in Table 2, univariate regression analysis demonstrated that BMI and Gal-9 were the potential predictors of NAFLD for RA patients (Odds ratio [OR]: 1.90, *p* < 0.05 and OR: 10.2, *p* < 0.05, respectively). Multivariate regression analysis also identified BMI and Gal-9 as significant predictors of NAFLD. Regarding the predictors of moderate-to-severe NAFLD in RA patients, univariate regression analysis identified Gal-9, sTIM-3, and FABP1 as the potential predictors (OR: 4.87, *p* < 0.05, OR: 4.30, *p* < 0.05 and OR: 6.23, *p* < 0.05, respectively). To establish the best model to predict the severity of NAFLD in RA, variables that were significant in the univariate regression analysis were further evaluated in the multivariate regression analysis. It revealed Gal-9 and sTIM-3 as significant predictors of severe NAFLD in RA. (OR: 5.02, *p* < 0.05 and 4.59, *p* < 0.05, respectively) (Table 3).

**Table 2:** Logistic regression analysis of laboratory parameters to predict RA patients with NAFLD.

Baseline variables	Univariate model			Multivariate model		
	OR	95%CI	*p* value	OR	95%CI	*p* value
Age, years	0.92	(0.84–1.01)	0.063			
Gender						
Male	ref.					
Female	0.61	(0.06–5.82)	0.669			
BMI	1.90	(1.15–3.14)	0.012	1.96	(1.10–3.52)	0.024
Gal-9	10.2	(1.14–92.3)	0.038	113.8	(1.14–169.4)	0.039
sTIM3	1.42	(0.30–6.81)	0.664			
FABP1	1.45	(0.32–6.80)	0.624			
FABP4	1.76	(0.36–8.46)	0.481			

Variables in multivariate model (backward: Conditional): gal-9 and BMI. Cut-off value: gal-9 (3.30 ng/mL); OR, Odds ratio; 95% CI, 95% confidence interval; gal-9, galectin-9; sTIM3, soluble T cell immunoglobulin and mucin-domain containing-3; FABP, fatty acid binding proteins.

**Table 3:** Logistic regression analysis of laboratory parameters to predict the severity of NAFLD in RA patients.

Baseline variables	Univariate model			Multivariate model		
	OR	95%CI	*p* value	OR	95%CI	*p* value
Age, years	1.02	(0.97–1.08)	0.482			
Gender						
Male	ref.					
Female	1.27	(0.22–7.29)	0.789			
BMI	1.10	(0.93–1.29)	0.274			
Gal-9	4.87	(1.12–21.1)	0.035	5.20	(1.10–24.6)	0.038
sTIM3	4.30	(1.08–17.2)	0.039	4.59	(1.05–20.1)	0.043
FABP1	6.23	(1.19–32.7)	0.031			
FABP4	1.12	(0.94–1.34)	0.213			

Variables in multivariate model (backward: Conditional): gal-9, sTIM3 and FABP1. Cut-off value: gal-9 (3.30 ng/mL), sTIM3 (4.2 ng/mL), FABP1 (1.40 ng/mL), OR, Odds ratio; 95% CI, 95% confidence interval; gal-9, galectin-9; sTIM3, soluble T cell immunoglobulin and mucin-domain containing-3; FABP, fatty acid binding proteins.

## Discussion

With the high prevalence of NAFLD among RA patients, it is an unmet need to find reliable biomarkers as a non-invasive approach to detect the emergence of NAFLD in them. This study showed that RA patients had significantly higher serum levels of Gal-9, sTIM-3, and FABP1 than HC participants. Gal-9, sTIM-3, and FABP1 were further significantly higher in RA patients with moderate-to-severe NAFLD than in those with none-to-mild NAFLD. Serum Gal-9 levels were positively correlated with NAFLD severity shown by sonography and serum levels of sTIM-3, FABP1, and FABP4, respectively, in RA patients. CRP levels and DAS28-ESR scores were also positively correlated with Gal-9, FABP1, and FABP4 in RA patients. Serum Gal-9 and FABP1 were significantly decreased in DMARDs-treated patients, paralleling the reduction of RA disease activity. The multivariate regression analysis revealed Gal-9 as a significant predictor of NAFLD development and Gal-9 and sTIM-3 as predictors of NAFLD severity. In the cell-based assay, Gal-9 could enhance lipid droplet accumulation in human hepatocytes through upregulating FABP1 expression. These findings suggest that Gal-9 and RA-related inflammation could be involved in the pathogenesis of RA-related NAFLD and Gal-9 level may be useful for predicting NAFLD development.

Given the high prevalence of NAFLD among RA patients (11, 12), early detection of NAFLD is key to the prevention of its long-term sequelae in these patients. To avoid the invasiveness and significant bleeding risk of liver biopsy (14), it is advisable to determine blood-based markers as a non-invasive NAFLD assessment. Gal-9, a multifunctional member of the galectin family, plays a crucial role in regulating immune reactions and inflammatory response (16, 17). Increasing evidence reveals that Gal-9 levels are elevated and correlated with disease activity in RA patients (19, 41, 42). In agreement with these findings (41, 42), serum Gal-9 levels in our RA patients were significantly higher than in healthy participants. Besides, serum Gal-9 levels were correlated with RA disease activity reflected by DAS28-ESR scores in our RA patients, as has been previously reported (41). TIM-3 is an immune-checkpoint molecule participating in the Gal-9/TIM-3 singling (42) and is involved in inflammatory bone erosion in RA (43). Resonated with these findings (43), serum sTIM-3 levels in our RA patients were not only significantly higher than in healthy participants but also significantly correlated with RA disease activity. Therefore, Gal-9 and TIM-3 probably play a significant role in the pathogenesis of RA. We surmise that serum Gal-9 and sTIM-3 levels may be related to NAFLD in RA patients, which has scarcely been explored.

Among the available non-invasive tools to evaluate NAFLD, liver ultrasonography is often used to estimate the severity of NAFLD. In the present study, we dichotomized RA patients into none-to-mild and moderate-to-severe groups based on NAFLD severity. RA patients with moderate-to-severe NAFLD had significantly higher levels of serum Gal-9 and sTIM-3 than those with none-to-mild NAFLD. Serum Gal-9 levels were also positively correlated with sTIM-3 levels and NAFLD severity gradings in our RA patients, supporting the findings that the Gal-9/TIM-3 signaling contributed to NAFLD in a murine model (24). Moreover, our results resonated with Fujita’s report that elevated levels of serum Gal-9 were associated with the emergence of liver fibrosis (44), a severe form of NAFLD. The involvement of Gal-9 in NAFLD pathogenesis may be related to the excessive production of Gal-9 by hepatic Kupffer cells and macrophages (45). Although APRI and FIB-4 scores have been used as additional noninvasive biomarkers to evaluate NAFLD severity (13), our results showed no significant difference in APRI or FIB-4 scores between RA patients with moderate-to-severe NAFLD and none-to-mild NAFLD.

In the present study, significantly higher levels of FABP1, but not FABP4, were observed in RA patients with moderate-to-severe NAFLD than in those with none-to-mild NAFLD. It is compatible with the finding that FABP1 is crucial to NAFLD development in a murine model (30, 31). Besides, this *in-vitro* cell-based assay demonstrated that Gal-9 could significantly enhance lipid droplet accumulation in human hepatocytes, and Gal-9 and FFA had a synergistic effect on the augmented expression of FABP1 expression. Our results support the findings that the FFA mixture could regulate FAPB1 expression that substantially trapped lipids in the cells (46). Similarly, serum Gal-9 levels were positively correlated with FABP1 levels in our RA patients, and serum Gal-9 and FABP1 significantly declined in patients after 12 months of DMARDs therapy, paralleling the decrease in RA disease activity. These observations suggest that Gal-9 may enhance lipid accumulation within human hepatocytes and thereby contribute to NAFLD development through upregulating FABP1 expression. However, these findings need to be further validated.

**Figure 5:**
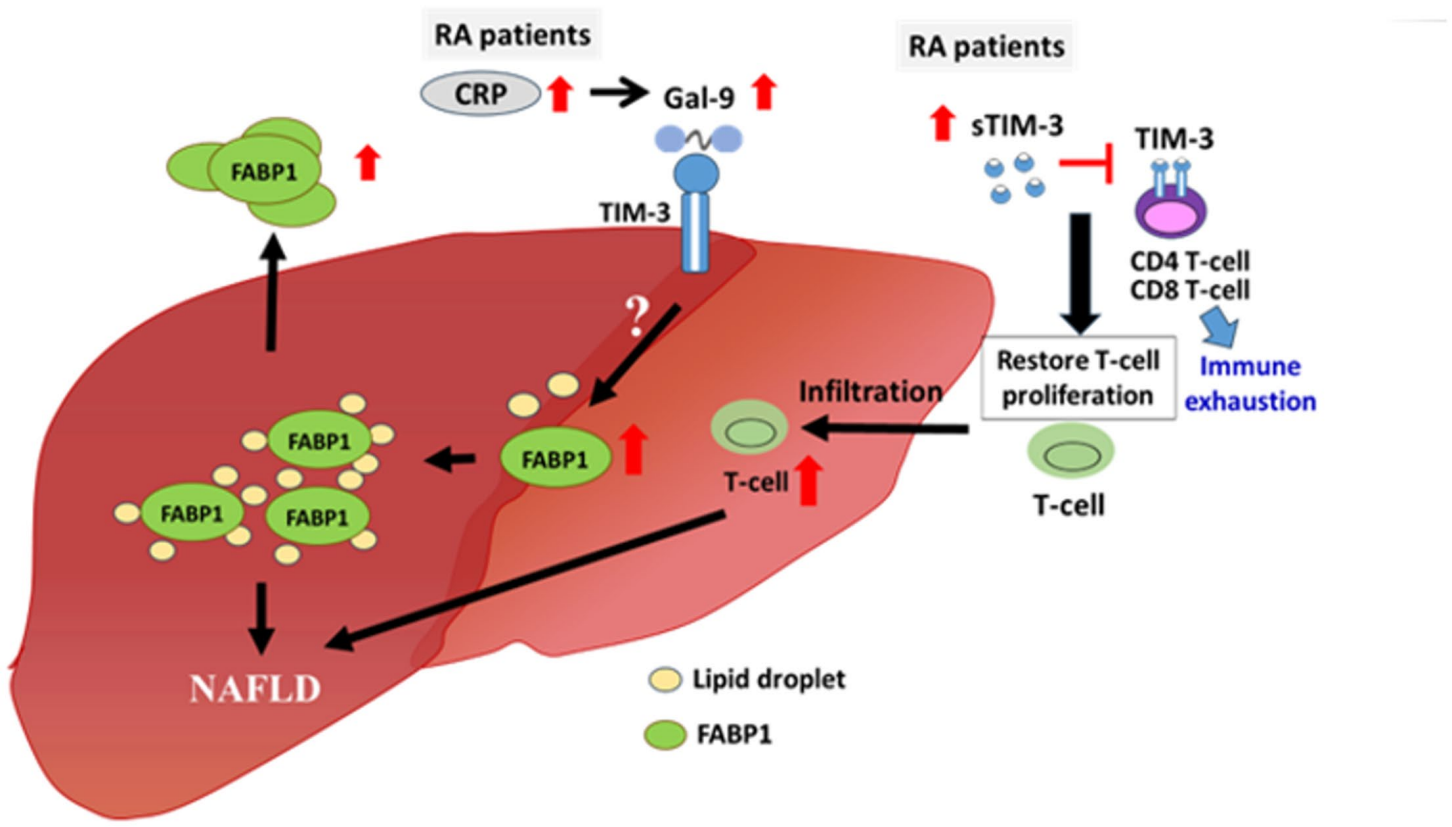
The potential pathogenic role of Gal-9 and sTIM-3 in RA-related NAFLD. Serum levels of Gal-9, sTIM-3 and FABP1 as well as systemic inflammatory parameters including CRP levels and DAS28-ESR scores were significantly elevated in RA patients and were even higher in those with moderate-to-severe NAFLD. Gal-9-induced FABP1 expression may be through the binding of TIM-3, resulting in accumulated lipid droplet in hepatocyte. Besides, the elevated levels of sTIM-3, which could block membrane-bound TIM-3 expressed on T cell and thus restored proliferation as well as activation of these immune cells. Gal-9, galectin-9; FABP, fatty acid binding protein; NAFLD, nonalcoholic fatty liver; RA, rheumatoid arthritis; CRP, C-reactive protein; DAS28-ESR, 28-joint disease activity score-erythrocyte sedimentation rate; sTIM-3, soluble TIM-3.

Increasing evidence indicates a pathogenic role of systemic inflammation in NAFLD (47, 48). In the present study, CRP levels and DAS28 scores, which reflected RA-related inflammation, were significantly and positively correlated with serum levels of Gal-9 and FABP1, which contribute to the development of NAFLD. We further demonstrated significantly higher CRP levels and DAS28 scores in RA patients with moderate-to-severe NAFLD than in those with none-to-mild NAFLD, supporting the link between inflammation and NAFLD. Besides, membrane-bound TIM-3 is highly expressed on peripheral T cells in RA (49) and plays a pivotal role in immune exhaustion (50). Our RA patients, particularly those with moderate-to-severe NAFLD, showed elevated levels of soluble TIM-3 (sTIM-3), which could block membrane-bound TIM-3 expressed on T cells and thus restore proliferation and activation of these immune cells (51). With the link between T cell inflammation and NAFLD progression (52), elevated sTIM-3 levels in our RA patients may contribute to NAFLD progression. Therefore, systemic inflammation and elevated levels of the Gal-9/TIM-3 may participate in the pathogenesis of RA-related NAFLD, as shown in the proposed model (Figure 5).

It is increasingly recognized that the use of DMARDs is implicated in the pathogenesis of NAFLD. Corticosteroids have the potential to disrupt lipid metabolism and induce insulin resistance, and prolonged or excessive use may raise the risk of triglyceride deposition in the liver (53). The use of cDMARDs, such as methotrexate (MTX), may also increase the risk of NAFLD in RA patients (9, 10). Our recent study revealed that hydroxychloroquine therapy was associated with a decreased risk of NAFLD (54). Resonated with the findings of JAKi-induced hyperlipidemia in RA patients (55), we demonstrated a non-significant increase in plasma levels of lipid profile after 6 months JAKi treatment. It is interesting that Gal-9 levels significantly declined in our RA patients treated with JAKi, suggesting that Gal-9 expression is provably enhanced through the JAK/STAT pathway (56). Since Gal-9 was associated with lipid droplet accumulation within hepatocytes in our study, JAKi may halt NAFLD progression through an inhibitory effect on Gal-9 and inflammation. Our results supported the findings conducted by Centa et al. that JAKi may reduce hepatic T cell infiltration and immune parameters in a murine model (52). Therefore, JAKi may potentially reduce NAFLD progression despite their hyperlipidemic effect. However, further extensive research is needed to explore the effects of JAKi on NAFLD development in RA patients.

The multivariate logistic regression analysis revealed BMI and Gal-9 as the significant predictors for the emergence of NAFLD. Our results support the findings of Loomis et al. that the risk of NAFLD increased linearly with BMI (57), and the report of Fujita et al. that a high Gal-9 level above 77.54 pg./mL was linked to a high probability of liver fibrosis (13). Besides, serum level of Gal-9 or sTIM-3 could be the significant predictors for the presence of moderate-to-severe NAFLD, with odds ratio (OR) of 5.20 or 4.59, respectively. Fujita et al. similarly revealed a significant OR of 3.90 for the ability of high Gal-9 levels to predict the progression of liver fibrosis (13). However, the use of cut-off level of serum Gal-9 in clinical practice still await further validation in other RA cohorts.

Despite the novel findings in this pilot study, there remain some limitations. Frist, there were no biopsy specimens available to prove NAFLD pathologically or grade the severity at the time of blood sampling for Gal-9, sTIM-3, FABP1, and FABP4. Additionally, it should be noted that certain animal models of RA may not entirely mirror the clinical and pathological characteristics of the human condition (58). Therefore, the choice of a model should be aligned appropriately with the specific objectives of the studies. Liver stiffness was not measured with transient elastography, which has high-performance characteristics for detecting severe fibrosis in NAFLD (59). All subjects enrolled in the present study are of Chinese ethnicity, and our findings may not be generalizable to other ethnic groups. The concomitant treatment with statin, corticosteroids, or csDMARDs in RA patients may also affect NAFLD (9, 10, 60, 61). Besides, with the small sample size of our RA patients, among whom the number of moderate-to-severe NAFLD cases was low, we could not provide a ROC-derived cut-off value of serum Gal-9 for predicting moderate-to-severe NAFLD. Although DM constitutes a significant risk factor for NAFLD, our results indicate no statistical difference between RA patients with moderate-to-severe NAFLD and those with none-to-mild NAFLD, possibly also attributable to the small sample size. Thus, our findings need to be confirmed by future large-scale studies enrolling more DMARDs-naïve RA patients.

This is the first to demonstrate that serum levels of Gal-9 and sTIM-3 were significantly elevated in RA patients and were even higher in those with moderate-to-severe NAFLD. Serum Gal-9 levels were positively correlated with sTIM-3 levels, FABP1 levels, NAFLD severity, or RA disease activity, respectively. Using both cell-based assay and human blood samples study, we revealed a pathogenic role of Gal-9 in the development of NAFLD through upregulation of FABP1. Our results also showed a positive correlation between CRP levels or RA activity scores and Gal-9 levels in RA patients. Besides, significantly higher CRP levels and DAS28 scores were observed in RA with moderate-to-severe NAFLD compared to those with none-to-mild NAFLD. We hypothesize that both Gal-9 singling pathway and systemic inflammation may contribute to NAFLD progression in RA patients, as shown in the proposed model (Figure 5). Serum Gal-9 levels could predict the development and severity of NAFLD in RA patients, although the causative role of Gal-9 and FABP1 in RA-related NAFLD still needs further investigation.

## Data availability statement

The raw data supporting the conclusions of this article will be made available by the authors, without undue reservation.

## Ethics statement

The studies involving humans were approved by Research Ethics Committee China Medical University & Hospital, Taichung, Taiwan. The studies were conducted in accordance with the local legislation and institutional requirements. The participants provided their written informed consent to participate in this study.

## Author contributions

P-KC: Conceptualization, Funding acquisition, Methodology, Writing – original draft, Investigation. W-FH: Formal analysis, Investigation, Methodology, Resources, Writing – review & editing. C-YP: Investigation, Methodology, Resources, Writing – review & editing. T-LL: Investigation, Methodology, Writing – review & editing. S-HC: Investigation, Methodology, Writing – review & editing. H-HC: Investigation, Methodology, Resources, Writing – review & editing. C-HC: Methodology, Writing – review & editing. D-YC: Conceptualization, Investigation, Project administration, Writing – original draft, Writing – review & editing.

## Figures and Tables

**Table:** 

ACR	American College of Rheumatology
Anti-CCP	anti-cyclic citrullinated peptide
DAS28	the 28-joint disease activity score
DM	diabetes mellitus
DMARDs	disease-modifying anti-rheumatic drugs
EULAR	European League Against Rheumatism
FABP	fatty acid binding protein
FFA	free fatty acid
FLI	fatty liver indicator
HC	health control
JAKi	Janus kinase inhibitors
MTX	methotrexate
NAFLD	non-alcoholic fatty liver disease
NK	natural killer
OA	oleic acid
PA	palmitic acid
RA	Rheumatoid arthritis
RF	rheumatoid factor
TC	total cholesterol
TG	Total glycerol
LDL	low-density lipoprotein
HDL	high-density lipoprotein
TNF	tumor necrosis factor
tsDMARDs	traditional synthetic DMARDs
US	Ultrasonography
Gal-9	galectin-9
sTIM-3	soluble a ligand of T cell immunoglobulin mucin-containing-molecule-3
