# Supplementary material for: Significant association of elevated serum galectin-9 levels with the development of non-alcoholic fatty liver disease in patients with rheumatoid arthritis
Source: Front Med (Lausanne). 2024 Feb 2;11:1347268. doi: 10.3389/fmed.2024.1347268 (PMC10869587; doi:10.3389/fmed.2024.1347268)
Supplement: Supplementary file 1 [file Table_1.docx]

**Supplementary Table 1.** Demographic data, clinical characteristics, laboratory findings, and the used medications in rheumatoid arthritis (RA) patients and Healthy control (HC)^#^

| **Characteristics** | **RA**  **(n=45)** | **HC**  **(n=11)** | **p-value** |
| --- | --- | --- | --- |
| Age at study entry, years | 60.9 ± 13.9** | 50.1 ± 10.5 | 0.020 |
| Female, n (%) | 37 (84.6%) | 10 (81.3%) | 0.482 |
| Disease duration, years | 9.54 ± 3.26 |  |  |
| Body mass index, kg/m^2^ | 24.9 ± 3.9 | 21.8 ± 1.3 | 0.008 |
| Total cholesterol, mg/dL | 201.6 ± 36.2 | 184.7 ± 34.2 | 0.109 |
| Triglyceride, mg/dL | 128.9 ± 104.5** | 60.3 ± 24.5 | 0.001 |
| LDL-C, mg/dL | 116.0 ± 29.2 | 109.2 ± 21.7 | 0.429 |
| HDL-C, mg/dL | 61.6 ± 15.7 | 64.5 ± 14.5 | 0.512 |
| Atherogenic index, (%) | 3.40 ± 0.89 | 2.93 ± 0.50 | 0.164 |
| RF-positivity, n (%) | 29 (64.4%) | NA |  |
| ACPA-positivity, n (%) | 26 (57.8%) | NA |  |
| C-reactive protein, mg/dL | 0.75 ± 1.13 | NA |  |
| DAS28 score at baseline | 3.53 ± 1.20 | NA |  |
| Concomitant corticosteroids, mg/day | 0.36 ± 0.71 | NA |  |
| Concomitant csDMARDs, n (%) |  |  |  |
| Methotrexate | 37 (82.2%) | NA |  |
| Hydroxychloroquine | 24 (53.3%) | NA |  |
| Sulfasalazine | 16 (35.6%) | NA |  |
| The use of bDMARDs, n (%) | 23 (51.1%) | NA |  |
| The use of JAK inhibitors, n (%) | 19 (42.2%) | NA |  |
| Comorbidities, n (%) |  |  |  |
| Hypertension | 14 (31.1%) | 0 (0.0%) | 0.048 |
| Diabetes mellitus | 5 (11.1%) | 0 (0.0%) | 0.571 |
| Current smoker, n (%) | 4 (8.9%) | 0 (0.0%) | 0.575 |

#Data were expressed as mean ± SD, number (%). NA: not applicable; ACPA, anti-citrullinated peptide antibodies; DAS28, the 28-joint disease activity score; HC, healthy control;RF, rheumatoid factor; LDL-C, low-density lipoprotein cholesterol; HDL-C, high-density lipoprotein cholesterol; csDMARDs, conventional synthetic disease-modifying anti-rheumatic drugs; bDMARDs, biological DMARDs; JAK, Janus kinase. Atherogenic index is the ratio of total cholesterol/HDL-C. Chi-square test was used to compare binary variables.

**p<0.01, ***p<0.001, moderate-severe in RA patients vs. none-mild NAFLD in RA patients as determined by Mann-Whitney test.
